# Effect of the dietary polyacetylenes falcarinol and falcarindiol on the gut microbiota composition in a rat model of colorectal cancer

**DOI:** 10.1186/s13104-018-3527-y

**Published:** 2018-06-27

**Authors:** Morten Kobaek-Larsen, Dennis Sandris Nielsen, Witold Kot, Łukasz Krych, Lars Porskjær Christensen, Gunnar Baatrup

**Affiliations:** 1 Department of Clinical Research, University of Southern Denmark, 5000 Odense, Denmark; 2 Department of Surgery, Odense University Hospital, 5000 Odense, Denmark; 3 Department of Food Science, Faculty of Science, University of Copenhagen, 1958 Frederiksberg, Denmark; 4 Department of Environmental Sciences, Aarhus University, 4000 Roskilde, Denmark; 5 Department of Chemistry and Bioscience, Faculty of Engineering and Science, Aalborg University, 9220 Aalborg Ø, Denmark

**Keywords:** Carrots, Rat model, Colorectal cancer, Polyacetylenes, Falcarinol, Falcarindiol, Microbiota

## Abstract

**Objectives:**

(3*R*)-Falcarinol (FaOH) and (3*R*,8*S*)-falcarindiol (FaDOH) have previously been shown to reduce the number of neoplastic lesions and the growth rate of polyps in the colon of azoxymethane (AOM) treated rats. Based on previous investigations, it appears that different mechanisms of actions are involved in the antineoplastic effect of FaOH and FaDOH. One mechanism of action may be related to the antibacterial effect of FaOH and FaDOH and thus their effect on the gut microbiota. This study aimed to determine the effect of FaOH and FaDOH on gut microbiota composition of AOM treated rats.

**Results:**

Azoxymethane treated rats were fed either a standard rat diet or a rat diet supplemented with FaOH and FaDOH. The gut microbiota of AOM-induced rats was determined by 16S rRNA gene-amplicon sequencing. Analysis of fecal cecum samples demonstrated a significant gut microbiota change in rats receiving standard rat diet supplemented with FaOH and FaDOH compared with the control group that only received the rat diet. Comparison of the gut microbiota of rats who developed large neoplasms in the colon with rats without large neoplasms showed that the gut microbiota was significantly different in rats who developed large colon neoplasms compared to rats with no macroscopic colon neoplasms.

## Introduction

Colorectal cancer (CRC) is a life-threatening disease with high incidence, morbidity, and mortality. Several lifestyle-related factors, such as diet, weight, heavy alcohol use, and physical inactivity, have all been linked to CRC [1], but during recent years, it has also become evident that gut microbiota (GM) dysbiosis influence development of colon cancer and benign tumors [2–4].

We have recently demonstrated that dietary supplements with (3*R*)-falcarinol [(3*R*,9*Z*)-heptadeca-1,9-dien-4,6-diyn-3-ol; FaOH] and (3*R*,8*S*)-falcarindiol [(3*R*,8*S*,9Z)-heptadeca-1,9-dien-4,6-diyn-3,8-diol; FaDOH], isolated from carrots, reduce the number of neoplastic lesions as well as the growth rate of the polyps in the colon of azoxymethane (AOM) treated rats. This suggest a preventive effect of FaOH and FaDOH on the development of CRC [5]. The inhibition of tumor growth was executed later, when AOM was no longer present in the rats. Hence, the observed inhibitory effect on the formation of neoplastic lesions of FaOH and FaDOH must be due to several mechanisms of action. One possible mechanism of action could be inhibition of the pleiotropic proinflammatory cytokines and their upstream NF-κB, signaling pathway, which is mandatory for neoplastic transformation and promotion [6]. Another mechanism could be changes in the intestinal microbiota. The intestinal microbiota has a symbiotic relationship with the host and the microbiota is responsible for the metabolism of otherwise non-digestible food sources, immune surveillance and protection of the barrier in the healthy individuals [2–4]. In addition, the microbiota of the intestine seems to be very important for the metabolism of chemical compounds influencing the host metabolome [7]. In this research note, we describe changes in the GM associated with FaOH and FaDOH supplementation in a rat model of CRC to elucidate a possible mechanism of action that to some extent can explain the preventive effect of these polyacetylenes on the development of CRC.

## Main text

### Materials and methods

#### Animal study

The animal study was approved by the central Animal Experimentation Inspectorate in Denmark (License no. 2015-15-0201-00708), and has previously been described in details [5, 8]. Male rats from the F344 strain with a certified health report were purchased from Charles River. The animals were 5 weeks old at the time of arrival. After 1 week of acclimatization, the rats were divided into 2 groups and started on the dedicated diets. The rats were fed on different diets for 2 weeks before the first injection with AOM at the age of 8 weeks [5, 8].

#### Rat diet

Powder/meal maintenance rat diet (Altromin 1321, Brogaarden Denmark) was used as standard diet for feeding the rats. Diet group 1 received standard rat diet supplemented with 7 µg FaOH/g feed and 7 µg FaDOH/g feed. The polyacetylenes FaOH and FaDOH were isolated from carrots by flash chromatography and preparative HPLC and identified by liquid chromatography tandem mass spectrometry (LC–MS/MS), NMR spectroscopy and optical rotation as described previously [5]. Diet group 2 only received standard rat diet. Because the purified FaOH and FaDOH (purity > 99%) was added to the diet in the form of an ethanol solution, the diet of the control group (group 2) was added the same amount of ethanol. Portions of 3.5 kg diet were prepared weekly for each of the two groups. The concentrations of FaOH and FaDOH in the rat diets were determined by LC–MS/MS before use. Diet group 2 was used as a negative control. No sign of degradation, oxidation or isomerization of FaOH and FaDOH was observed during the animal study as well as no significant differences in the content of FaOH or FaDOH in the weekly prepared diet [5].

#### Autopsy procedures

**Fig. 1:**
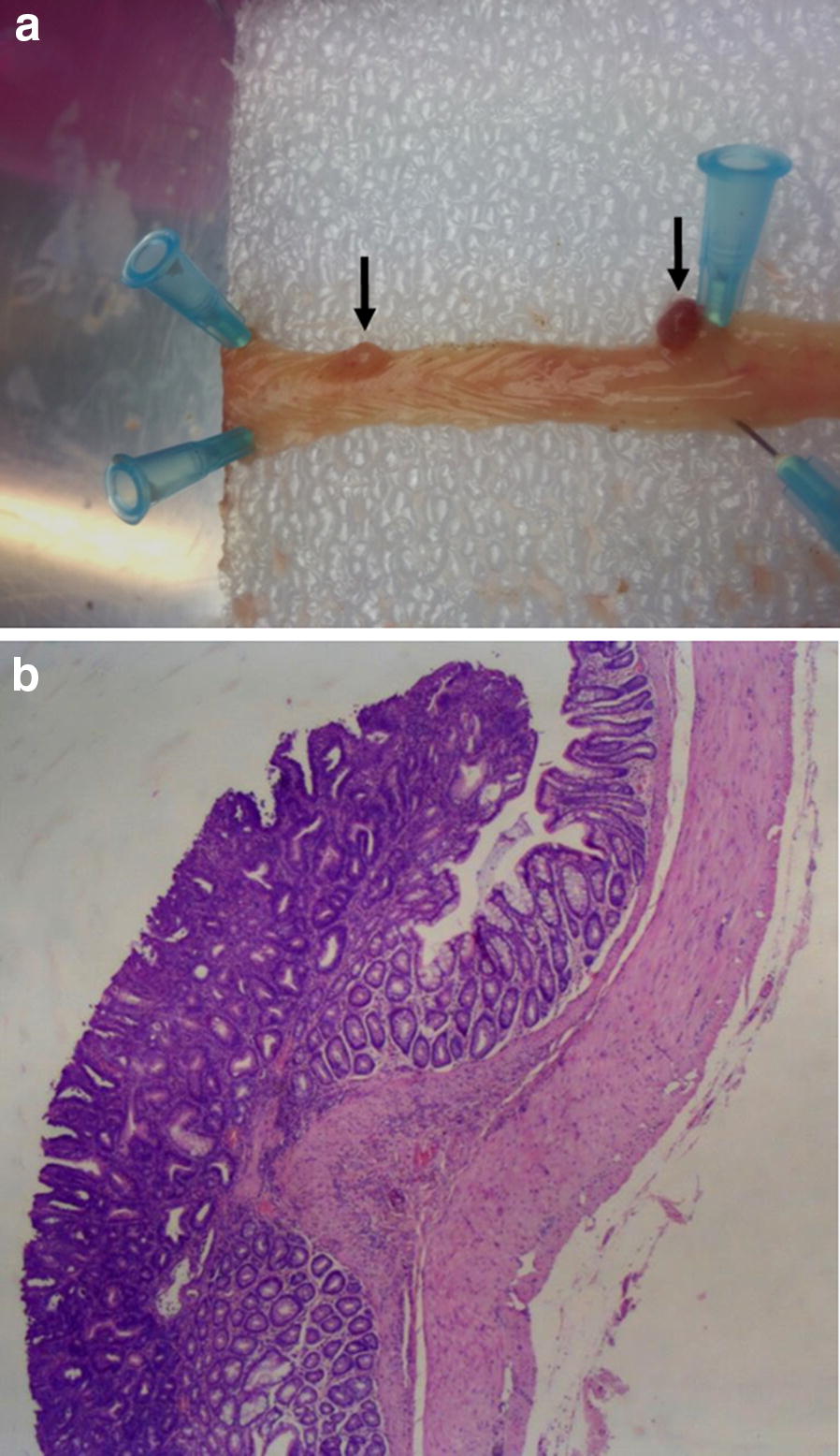
**a** Macroscopic appearance of neoplasms (black arrows). **b** Histology of adenoma. Hematoxylin and eosin were used for staining

The rats were euthanized 18 weeks after the first AOM injection and autopsied to examine for macroscopic alterations. The animals were killed by cervical dislocation, after they had been anaesthetized with isoflurane inhalation. Immediately after death, luminal content was collected from cecum and stored at − 80 °C until analysis. Macroscopic findings were confirmed by histological analyses on hematoxylin and eosin stained sections (Fig. 1) as described previously [5].

#### Analysis of microbiota

Total genomic DNA was extracted from the fecal samples using the MoBio Powersoil kit (Qiagen) following the instructions of the manufacturer with the inclusion of an additional bead-beating step to ensure efficient bacterial cell lysis. Gut prokaryotic composition was determined by NextSeq-based 16S rRNA gene-amplicon sequencing of the V3 region amplified using primers [9] designed with adapters for the Nextera Index Kit^®^ (Illumina, CA, USA): NXt_338_F: 5′-TCG TCG GCA GCG TCA GAT GTG TAT AAG AGA CAG ACW CCT ACG GGW GGC AGC AG-3′ and NXt_518_R: 5′-GTC TCG TGG GCT CGG AGA TGT GTA TAA GAG ACA GAT TAC CGC GGC TGC TGG-3′. Amplification profile (1st PCR), barcoding (2nd PCR), amplicon library purification and sequencing (Illumina NextSeq-platform, 2 × 150 cycles, MID output) were performed as previously described [10]. The raw dataset containing pair-ended reads with corresponding quality scores were merged and trimmed [11]. Quantitative Insight Into Microbial Ecology (QIIME) open source software package [12] (1.7.0, 1.8.0, 1.9.0) was used for subsequent analysis. Purging the dataset from chimeric reads and constructing de novo Operational Taxonomic Units (OTU) was conducted using the UPARSE pipeline [13]. The green genes (13.8) 16S rRNA gene collection was used as a reference sequences. Three samples were excluded from the analysis due to low read number. UniFrac distance matrices were generated with the Jackknifed Beta Diversity workflow based on 10 distance metrics calculated using 10 subsampled OTU tables and projected using non-metric multidimensional scaling. The number of sequences taken for each jackknifed subset was set to 85% of the sequence number within the most indigent sample (25,000).

#### Statistical analysis

Analysis of Similarities (ANOSIM) was used to evaluate group differences based on weighted, unweighted UniFrac distance matrices. Alpha diversity measures expressed with an observed species (sequence similarity 97% OTUs) value were computed for rarefied OTU tables using the alpha rarefaction workflow. Differences in alpha diversity were determined using a *t*-test-based approach employing the non-parametric (Monte Carlo) method (999 permutations) implemented in the compare alpha diversity workflow. The differences in taxa abundance between categories were estimated with a statistic framework: analysis of composition of microbes (ANCOM) based on non-normalised OTU-table summarized to the species level [14].

## Results and discussion

**Fig. 2:**
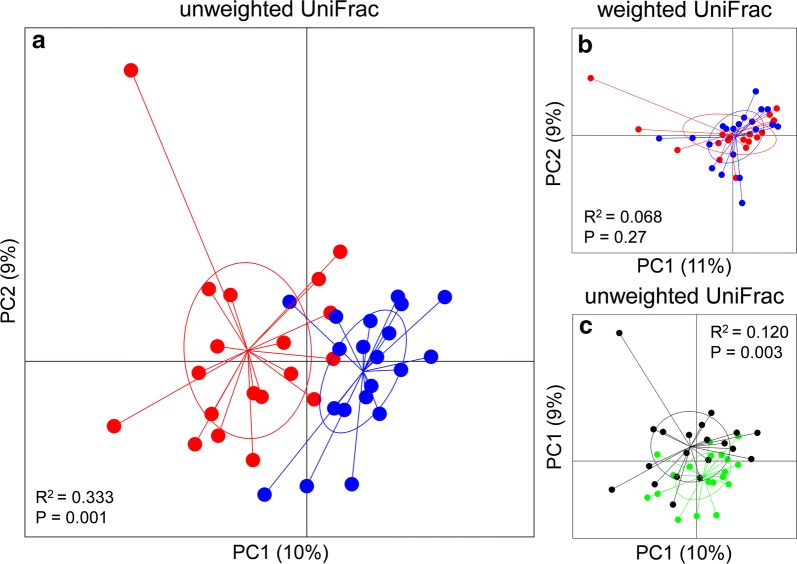
Inclusion of FaOH and FaDOH in the diet leads to significant changes in the composition of the low-abundant taxa in the rat colon, which also influence neoplasia occurrence. Principal Coordinate Analysis plot depicting **a** unweighted and **b** weighted UniFrac distance metrics based on 16S rRNA gene (V3 region) amplicon sequencing of fecal content of rats fed standard diet (red dots) and diet containing FaOH and FaDOH (blue dots). **c** Unweighted UniFrac distance metrics based on 16S rRNA gene (V3 region) amplicon sequencing of fecal content of rats developing neoplasms (black dots) or not (green dots)

**Fig. 3:**
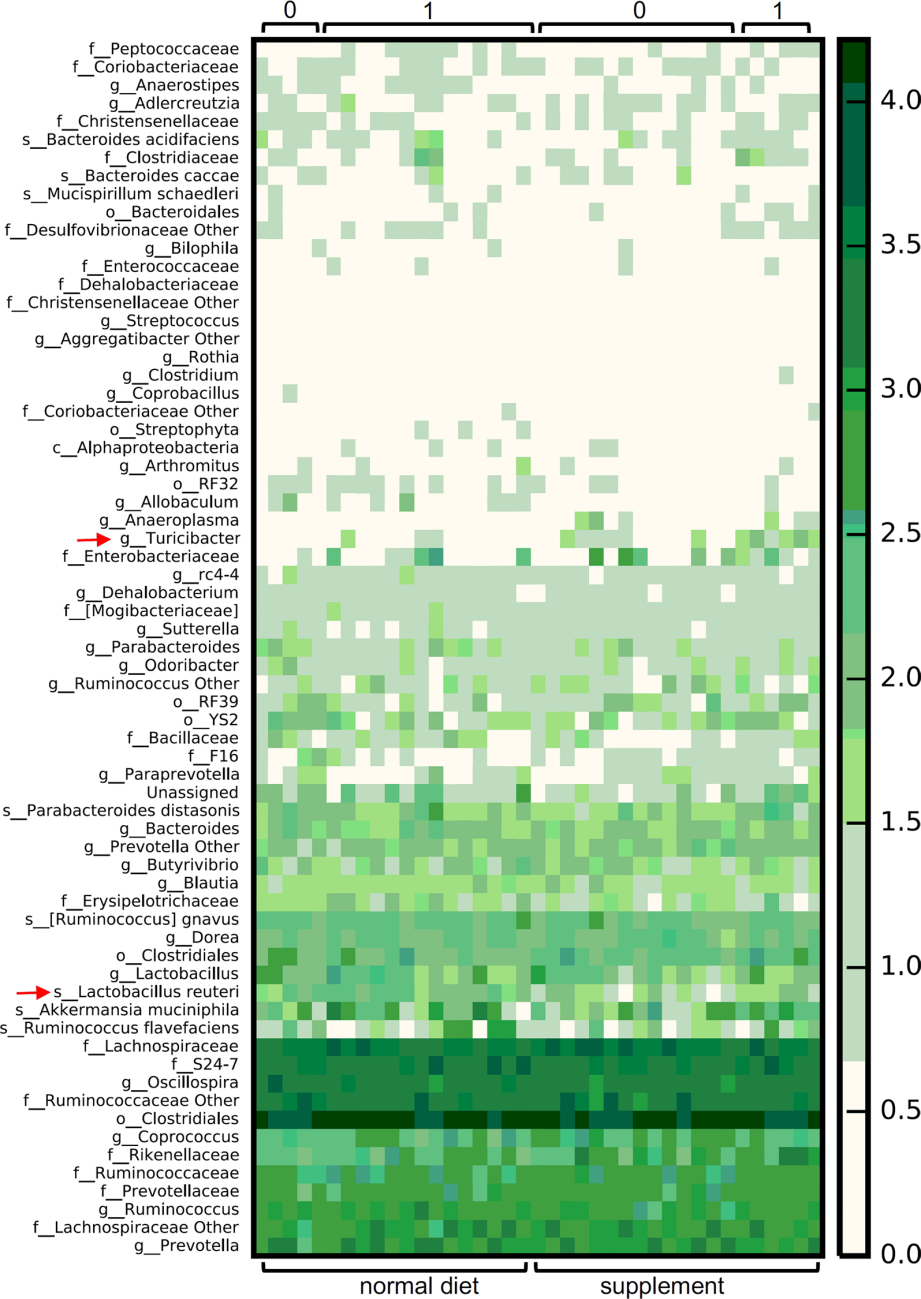
Heatmap illustrating fecal abundance of taxa characterized by16S rRNA gene amplicon sequencing. Occurrence of *Lactobacillus reuteri* is significantly different between rats receiving standard diet supplemented with FaOH and FaDOH vs. standard diet while the occurrence of *Turicibacter* is significantly different between rats with and without macroscopic colon neoplasms (ANCOM, P < 0.05). 0 = no neoplasia and 1 = neoplasia. Red arrows indicate the position of *Lactobacillus reuteri* and *Turicibacter*, respectively, in the heatmap

Inclusion of FaOH and FaDOH in the rat diets led to pronounced changes in composition of the less-abundant members of the GM as seen from unweighted UniFrac distance metrics (Fig. 2a, P = 0.001). Weighted UniFrac distance metrics-based analysis did on the other hand not reveal systematic differences between the two diets indicating that the relative abundance of the dominant species in the rat gut are not significantly influenced by inclusion of FaOH and FaDOH in the diet (Fig. 2b, P = 0.27). The majority of rats fed the FaOH and FaDOH supplemented diet, however, did not develop neoplasms as described previously [5] and as shown in Fig. 3. Furthermore, it was found that rats developing neoplasms had a GM differing from rats that did not develop neoplasms (Fig. 2c, unweighted Unifrac distance metrics, P = 0.003). Analysis of communities of microbes (ANCOM) showed that inclusion of FaOH and FaDOH in the diet (diet 1) led to a decrease in the prevalence of an OTU assigned to *Lactobacillus reuteri* (Fig. 3). Higher prevalence of *Turicibacter* was observed in the GM of rats not developing neoplasms (Fig. 3). The results of the present rat study therefore strongly indicate that FaOH and FaDOH have an effect on the GM, which might reduce the incidence of neoplastic lesions. The polyacetylenes FaOH and FaDOH have previously been shown to have antibacterial effects on both Gram-positive and Gram-negative bacteria as well as antimycobacterial effects [15–20]. However, the antibacterial effects of FaOH and FaDOH occurs at or above 10 μg/ml [15, 16, 18, 19], which is higher than the concentrations of the two compounds supplemented to the rat feed (7 µg/g feed) in this study. The results, however, indicate that at even sub-inhibitory concentrations these compounds are able to influence GM composition, possibly by influencing growth rate of some species, but not all.

Azoxymethane is not the final carcinogenic metabolite, but it needs further stepwise activation in order to induce neoplastic transformation. The process of activation has not yet been fully clarified but it includes a hydroxylation step mediated by cytochromes P450 in the liver to methylazoxymethanol (MAM). After excretion of MAM via the bile to the intestine, further metabolization is promoted by the colonic microbiota to methyldiazonium. This transformation of AOM seems to be necessary before AOM can exert its colonotropic mutagenicity [21]. The changes in the composition of the GM observed in this study could affect the susceptibility of the carcinogens on the colonic mucosa altering their colonotropic mutagenicity. One possible explanation for this is that the changed composition of the GM inhibits the conversion of MAM to methyldiazonium, which is mainly responsible for the genetic alteration in the mucosal cells [22]. Alternatively, the changed composition of the GM affects the host immune homeostasis lowering the inflammation developed during carcinogenesis [23]. Consequently, the antineoplastic effect of FaOH and FaDOH is not only caused by their cytotoxic and antiinflammatory effect, but may also be due to the changes they induce in the GM.

The composition of low abundant GM members seems to correlate with the observation of microscopic alterations vs. macroscopic alterations of the colonic submucosa (Figs. 2 and 3). In this model of CRC, with a latency time of 18 week, the neoplasms were mostly adenomas with high-grade dysplasia. Microscopic alterations refer to no macroscopic visual alterations. This may be important in understanding of the carcinogenesis process of large neoplasms and the preventive effect of the dietary polyacetylenes FaOH and FaDOH on the development of CRC.

In conclusion, this study revealed that FaOH and FaDOH, which have previously been shown to inhibit the formation of neoplastic tumors in the colon in a rat model of colon cancer, affect the composition of low abundant GM members, which in turn is associated with a reduced formation of macroscopic neoplasms. Thus, the present investigation has shown that changes in the GM may play an important role in the preventive effect of FaOH and FaDOH towards neoplastic transformation in the colon.

## Limitations

Chronic infection and inflammation contributes to CRC, although there is growing evidence that the GM play an important role in the progression of this disease. Even though microbiota-based cancer prevention, diagnosis, and therapy in humans are beginning to emerge, we still need more information about the microbiota composition to identify, which changes in the GM that may result in a preventive effect towards CRC in humans as well as in animals. Consequently, we are not able to conclude, whether the significant changes of the low abundant GM members in the microbiota of rats receiving FaOH and FaDOH in the diet compared with the control group, are essential, and thus contribute to an explanation to the preventive effects of FaOH and FaDOH towards CRC in the AOM treated rats.

## Abbreviations

AOM: azoxymethane
CRC: colorectal cancer
GM: gut microbiota
FaOH: falcarinol
FaDOH: falcarindiol
MAM: methylazoxymethanol
OUT: operational taxonomic units
